# SERS Performance of Ti_3_C_2_T*_x_* MXene-Based Substrates Correlates with Surface Morphology

**DOI:** 10.3390/ma17061385

**Published:** 2024-03-18

**Authors:** Farnoush Salehtash, Adriana Annušová, Anastasiia Stepura, Yaryna Soyka, Yuriy Halahovets, Monika Hofbauerová, Matej Mičušík, Mário Kotlár, Peter Nádaždy, Paweł Albrycht, Peter Šiffalovič, Matej Jergel, Mária Omastová, Eva Majková

**Affiliations:** 1 Institute of Physics, Slovak Academy of Sciences, Dúbravská cesta 9, 845 11 Bratislava, Slovakia; farnoush.salehtash@savba.sk (F.S.); adriana.annusova@savba.sk (A.A.); monika.hofbauerova@savba.sk (M.H.); peter.nadazdy@savba.sk (P.N.); peter.siffalovic@savba.sk (P.Š.); matej.jergel@savba.sk (M.J.); 2 Centre for Advanced Materials Application, Slovak Academy of Sciences, Dúbravská cesta 9, 845 11 Bratislava, Slovakia; 3 Polymer Institute, Slovak Academy of Sciences, Dúbravská cesta 9, 845 41 Bratislava, Slovakia; anastasiia.stepura@savba.sk (A.S.); upolyaso@savba.sk (Y.S.); upolmmic@savba.sk (M.M.); maria.omastova@savba.sk (M.O.); 4 Centre for Nanodiagnostics of Materials, Slovak University of Technology in Bratislava, Vazovova 5, 812 43 Bratislava, Slovakia; mario.kotlar@stuba.sk; 5 Institute of Electrical Engineering, Slovak Academy of Sciences, Dúbravská cesta 9, 841 04 Bratislava, Slovakia; 6 Institute of Physical Chemistry, Polish Academy of Sciences, Kasprzaka 44/52, 01-224 Warsaw, Poland; pawel@sersitive.eu

**Keywords:** surface-enhanced Raman scattering, MXene, Rhodamine B, paper substrates, vacuum-assisted filtration

## Abstract

The surface-enhanced Raman scattering (SERS) properties of low-dimensional semiconducting MXene nanoflakes have been investigated over the last decade. Despite this fact, the relationship between the surface characteristics and SERSing performance of a MXene layer has yet to be comprehensively investigated and elucidated. This work shows the importance of surface morphology on the overall SERS effect by studying few-layer Ti_3_C_2_T*_x_* MXene-based SERS substrates fabricated by vacuum-assisted filtration (VAF) and spray coating on filter paper. The VAF deposition results in a dense MXene layer suitable for SERS with high spot-to-spot and substrate-to-substrate reproducibility, with a significant limit of detection (LoD) of 20 nM for Rhodamine B analyte. The spray-coated MXenes film revealed lower uniformity, with a LoD of 50 nM for drop-casted analytes. Moreover, we concluded that the distribution of the analyte deposited onto the MXene layer is affected by the presence of MXene aggregates created during the deposition of the MXene layer. Accumulation of the analyte molecules in the vicinity of MXene aggregates was observed for drop-casted deposition of the analyte, which affects the resulting SERS enhancement. Ti_3_C_2_T*_x_* MXene layers deposited on filter paper by VAF offer great potential as a cost-effective, easy-to-manufacture, yet robust, platform for sensing applications.

## 1. Introduction

Surface-enhanced Raman spectroscopy (SERS) is an ultrasensitive method which enables simple and reliable identification of a small quantity of molecules. SERS analysis is non-destructive, fast, with high accuracy, and is widely explored for chemical and biological sensing and detection. The signal enhancement is attributed to the electromagnetic and chemical mechanisms. The electromagnetic mechanism relies on the generation of localized surface plasmons leading to a significant enhancement of the incident electromagnetic field that occurs preferentially at hot spots of the plasmonic materials, which are typically noble and coinage metals (e.g., Au or Ag) with nanoscale surface roughness. The electromagnetic mechanism provides high sensitivity. An enhancement factor (EF) up to 10^15^ could be achieved allowing single-molecule detection [1,2]. The chemical mechanism is related to the interaction between the analyte molecules and the SERS substrate surface such as charge transfer processes, resonant energy transfer, or modification of the electronic structure of the analyte molecule. Chemical enhancement strongly depends on the type of analyte and its interaction with the SERS substrate’s surface. Recently the research has been focused on new noble metal-free SERS substrates based on semiconducting materials, often in the form of low dimensional nanomaterials (nanoparticles, nanosheets). The advantages of 2D nanomaterials are lower fabrication costs, the possibility to tune their electronic properties for SERS application, and biocompatibility. The present strategy to improve the SERS sensitivity relies on the charge transfer resonance. The large specific surface of 2D nanomaterials is favorable for formation of surface atom and analyte molecule complexes and increases photoinduced charge transfer in comparison to the 3D materials [3,4,5].

Various strategies have been developed to prepare novel semiconducting materials with excellent SERS performances by optimizing their morphology, chemical composition, crystallinity, and defect formation. The ultrasensitive performance was reported for graphene and graphene oxide [6,7,8], where for N-doped graphene an EF of up to 10^11^ for Rhodamine B (RhB) was achieved. Semiconducting metallic oxide nanoobjects such as TiO_2_ [9] and Nb_2_O_5_ [10], oxygen-doped metallic dichalcogenides [11], organic heterostructures [12], and hybrid metal organic perovskites [13] have all been studied as promising SERS materials. Recently, MXene nanoflakes (see, e.g., recent review [14]) have been analyzed as novel promising candidates for SERS substrates.

MXenes are classes of 2D material with the general formula M_n+1_X_n_T*_x_*, where M is the transition metal and X is carbon/nitrogen, and T*_x_* represents surface termination groups (O, OH, F). MXenes are typically derived from layered transition metal carbides or nitrides known as MAX phases by selective etching process. MXenes exhibit nearly metallic properties such as an abundant density of states near the Fermi level, high electrical conductivity (in the 10^4^ S cm^−1^ range), and high carrier density at 3.8 × 10^22^ cm^−3^ [15,16]. Besides light absorption in the UV and visible region, some MXenes exhibit increased optical absorption in a wide range of the NIR region. The plasmonic properties of MXenes were anticipated by Maleski et al. [17], and up to now, they were reported by several authors (see, e.g., recent review [18]).

The first reports on the application of Ti_3_C_2_T*_x_* MXenes for SERS refer to the Ti_3_C_2_T*_x_* MXene hybrids with noble metal plasmonic nanoparticles Ag, Au, and Pt utilizing plasmonic as well as chemical enhancement [19,20]. For dyes, the EF up to 10^6^–10^8^ was reported for nanoparticle–MXene hybrid substrates. Besides dyes, MXene–noble metal hybrids have been successfully applied for detection of various chemicals such as chlorpromazine with a limit of detection (LoD) of 3.92 × 10^−11^ M [21]. Liu et al. [22] presented a MXene-based platform which could be applied as a sensor in clinical diagnosis and monitoring of the biological environment for the detection of biomolecules such as adenine and dopamine with a LoD of 10^−8^ M. Flexible hybrid MXene SERS substrates for pesticide detection on fruit were successfully developed by Xiong et al. [23].

Sarycheva et al., in their pioneering work [24], presented the SERS performance of spray-deposited Ti_3_C_2_T*_x_* on silicon and SiO_2_ substrates, with an EF for dye molecules of up to 10^6^. Here, the synergy of both electromagnetic and chemical enhancement mechanisms was suggested. Up to now, several MXene-based SERS substrates prepared by various deposition procedures have been published. The EFs in the micromolar range were reported for Ti_3_C_2_T*_x_* [25], Nb_2_CT*_x_* [26], and Ta_2_CT*_x_* [27] MXene SERS substrates and various dyes as analytes. Increased sensitivity was observed for MXenes prepared by specific procedures. For SERS substrates based on highly crystalline Ti_3_C_2_T*_x_* monolayer nanosheets, an EF of 10^8^ was observed for environmental pollutants [28]. For Ti_3_C_2_T*_x_* prepared by a modified procedure deposited on silicon LoDs, up to 10^−8^ M for dye molecules such as Crystal Violet, Rhodamine 6G, and Malachite green were reported [29]. Here, the potential for enhancement up to single-molecule detection relying on the synergy between the resonance Raman and charge-transfer transitions of Ti_3_C_2_T*_x_* monolayer nanosheets was suggested. Soundiraraju et al. applied Ti_2_NT*_x_* MXenes with higher charge carrier concentrations deposited on paper, silicon, and glass as SERS substrates for Rh6G detection [30]. The EF of 10^12^ was observed for the paper substrate, whereas for the other substrates an EF of 10^3^ was observed for several dyes. Recently, the SERS substrates of highly conductive V_4_C_3_T*_x_* and V_2_CT*_x_* MXenes with LoD of 10^−7^ M for dye analyte were published [31]. For bimetallic Ti-V-C highly crystalline MXene nanosheets, the femtomolar-level detection limit for R6G was reported recently [32].

The published experimental data for MXene-based SERS substrates and dyes as analyte, supported by density functional theory calculations, confirm the dominance of chemical mechanisms for Raman enhancement. Limbu et al. [25] estimated a 100-fold increase in the enhancement factors resulting from chemical mechanism compared to that of the electromagnetic one. The dominance of the chemical enhancement could also be supported by the observed red shift in the Raman spectra for analytes on MXene in comparison to the free molecules. The chemical enhancement involves charge transfer and molecular orientation effects at the MXene surface. The surface functional groups of MXenes can support the adsorption of the analyte and facilitate charge transfer between the analyte and the MXene resonant energy transfer, or modification of the electronic structure of the analyte molecule.

Dyes are used as Raman probes for comparisons of the SERS performance for substrates prepared by different deposition techniques and/or various types of MXenes. In recent years, papers presenting high sensitivity of MXene SERS substrates for various analytes such as proteins, environmental pollutants, pharmaceuticals, metabolites of pharmaceuticals, and/or volatile organic compound have been published as well. Detection limits up to 10^−11^ M were reported [27] for several environmental pollutants such as azo dyes, trichlorophenol, and bisphenol A for highly crystalline Ti_3_C_2_T*_x_* MXenes. Peng et al. [33] published high SERS sensitivity with an LoD of up to 5 × 10^−9^ M for the SARS-CoV-2 spike protein for Ta_2_CT*_x_* MXene substrates. This enhancement could enable real-time monitoring and early warning of novel coronavirus. Recently, Peng reported the ultrahigh sensitivity for chloramphenicol detection for the novel Nb_2_C-based multiporous MXenes stacking structure [34]. MXenes are promising materials in SERS-based sensors too. In [35], the authors present for the first time the Ti_3_C_2_T*_x_* MXene-based sensor for salicylic acid detection. The adsorption of the salicylic acid molecules and the formation of MXene salicylic acid complexes were experimentally confirmed. The experimentally evaluated enhancement factor can vary from 220 to 60 when different excitation wavelengths are applied. The application of MXenes in a microfluidic gas sensing structure revealed multiple detecting ability and high sensitivity with an LoD in the range of 10–50 ppb as was demonstrated by Yang et al. [36].

In summary, the detection limit in micromolar ranges is mostly reported for dye analytes on MXene SERS substrates prepared by standard processes. At present, the research is aimed to increase their SERS sensitivity up to nano- or femtoM levels. The enhancement strategies focus mostly on new MXenes composition (e.g., double metal MXenes) and/or specific processing of few-layer MXene nanoflakes such as improvement in their crystalline structure, suppressing the oxidation of the nanoflakes, and modification of the surface functional groups. The published results point also at the strong effect of the deposition technique applied for the preparation of MXene substrates on their SERS performance. Various methods such as spin coating, drop casting, spray coating, and, recently, vacuum-assisted filtration (VAF) have been reported for assembling the MXene nanoflakes. Here, VAF could be an effective approach for preparation of a dense MXene layer of MXene nanosheets oriented mostly parallel with the substrate surface. The subsequent evaporation of water generates capillary compression force perpendicular to the nanosheets, which results in dense arrangement of the nanoflakes in the deposited layer [37].

Another important point in SERS analysis is the adsorption efficiency of analytes as more molecules of the analyte adsorbed on the substrate surface will result in stronger Raman signals [2]. It is already well established for noble metal SERS substrates that efficient analyte manipulation improves the SERS detection. This is especially important for analytes with no specific affinity for the particular SERS substrate. Here, the various molecular enrichment strategies are developed to increase the sensitivity and specificity of SERS-based detection methods, see, e.g., [2]. They can be based on approaches such as chemical modification, electrophoretic deposition, microfluidics, application of the electric field, and vacuum-assisted filtration (VAF) [37,38] for facilitating the deposition of analyte. For noble-metal-free substrates, the development of simple, rapid, and efficient strategies to achieve molecular enrichment is still a challenge. For MXenes, strategies such as the surface functionalization for particular analyte molecules, and modification of the surface morphology of the MXenes substrate by etching, annealing, and nanoparticle depositions could be considered. Different MXene compositions could exhibit various affinities towards different analytes, and the proper choice of MXene could enhance the adsorption of the analyte molecules. The VAF method was recently applied for analyte deposition onto vanadium carbide MXene spray-coated substrates. Here, the detection with sensitivity of 5 nM level for Rhodamine 6G (Rh6G) was reported [31].

In this work, we present few-layer Ti_3_C_2_T*_x_* MXene-based SERS substrates fabricated by VAF on filtration paper. The RhB was used as an analyte. The VAF deposition enables us to prepare a dense MXene layer suitable for SERS with high uniformity in a simple method without any complicated deposition equipment. In order to bring the maximum amount of the analyte in the close vicinity of MXenes, we relied on molecular enrichment applying the VAF method too. By applying this preparation process, an LoD of 20 nM was achieved. The substrates revealed high spot-to-spot and also substrate-to-substrate reproducibility. The spray-coated MXenes film on filtration paper, prepared for comparison, revealed lower uniformity, and for the drop-casted analyte an LoD value of 50 nM was observed. A strong effect of the MXene aggregates on the distribution of the drop-cast analyte was demonstrated and analyzed. Conclusions were drawn on the importance of surface morphology of the SERS layer on the overall SERS effect.

## 2. Materials and Methods

### 2.1. Synthesis and Analysis of 2D MXene Nanoflakes

The multilayered Ti_3_C_2_T*_x_* MXene (ML MXene) nanoflakes were obtained in the form of a water-based paste from Drexel University (Philadelphia, PA, USA). Details of the delamination reaction are described elsewhere [39]. For the delamination reaction, the mixture of ML MXenes with LiCl was stirred overnight on a magnetic stirrer and then centrifuged numerous times using deionized water to obtain a solution of the few-layered (FL) MXene nanoflakes.

The FL MXene nanosheets were characterized using a JEOL JEM ARM200CF high-resolution transmission electron microscope (HRTEM, STEM) equipped with an image corrector operated at 80 kV and 200 kV. The nanosheets from the solution were dispersed on lacey carbon supported copper mesh grid. STEM images were recorded with ADF and BF detectors.

The morphology of MXene nanoflakes was analyzed by SEM and AFM. For analysis, a single layer of FL MXenes was deposited on the Si substrate by the Langmuir-Schaefer method. AFM measurements were performed using Dimension Edge Bruker (Bruker, Berlin, Germany) in tapping mode. The presence of FL MXenes was confirmed by Grazing incidence XRD (GIXRD) of the FL MXenes layer drop-casted on the Si substrate. The measurement was performed in coplanar geometry (diffractometer setup D8 Discover SSS with rotating anode (Bruker AXS, Karlsruhe, Germany) with CuKα radiation at 40 kV/300 mA exposure and a fixed incident angle of 1°.

The chemical state of the prepared FL MXenes was inspected by XPS using a NEXSA-G2 system (Thermo Fisher Scientific, Brno, Czech Republic) equipped with a micro-focused, monochromatic Al Kα X-ray source (1486.68 eV). An X-ray beam was focused on a 400 μm spot on the sample surface. FL MXenes were drop-casted on a pre-cleaned silicon wafer. The spectra for the survey were acquired in constant analyzer energy mode with a pass energy of 200 eV. The high-resolution spectra were collected with a pass energy of 50 eV. Charge compensation was achieved with an Ar flood gun system. The Thermo Scientific Avantage software (version 6.6.0, Thermo Fisher Scientific) was used for digital acquisition and data processing.

### 2.2. Synthesis and Analysis of MXene-Based SERS Substrates

The MXene nanosheets were assembled into MXene film by VAF method on filtration paper. The filtration paper was purchased from Fisher Scientific, type FISHERBRAND 15A (cat.no. 3122-3153). The paper was secured to the opening of the glass Buchner flask connected to a membrane vacuum pump. A volume of 5 mL of MXene solution of a concentration 0.63 mg mL^−1^ was pipetted onto the paper surface, then the vacuum pump was turned on. The pumping lasted approximately 5 h, until the majority of the paper appeared dried (the details are given in Supplementary Materials, Figure S1). Additionally, MXene films sprayed on filtration paper and/or glass were prepared. The glass substrates were cleaned with acetone, isopropanol, and water by ultrasonication, dried under N_2_ gas flow, and put under a UV lamp for 10 min before use. MXene solution of 3.4 mg mL^−1^ concentration was sprayed over the glass substrates over 10 times using an airbrush pistol (Airbrush HS07-3/HS08-3). After each spraying, the layer was left to air dry before the next spraying. For comparison of the prepared substrates to commercial ones, we procured silver-based ITO SERS substrates (SERSitive, Warsaw, Poland).

Surface/cross section morphology of the prepared MXene SERS substrates was characterized by high-resolution Scanning Electron Microscopy (SEM), Thermo Fisher Scios 2 LoVac. Images were recorded using ETD detector in secondary electron mode at 10 kV accelerating voltage and 7 mm working distance. We analyzed different areas of the MXene SERS substrate and pristine paper for comparison. To compensate charging effects during cross section measurements we sputtered samples with 5 nm thick Au layer. The chemical composition of the samples was analyzed by EDS using Thermo Scientific UltraDry EDS spectrometer with active area of 60 mm^2^ at 10 kV electron accelerating voltage.

The distribution of the analyte over the SERS substrates was inspected by Confocal Laser Scanning Microscopy (CLSM). Measurements were performed by Zeiss LSM 900 using a 40× magnification objective (Carl Zeiss 40×/0.55 NA) and a filter for RhB (Exc/Em = 543/565 nm).

For paper-based SERS substrates, 50 μL of RhB was deposited by the VAF (described above) which lasted 3 h. For sprayed SERS substrates, 50 μL of RhB was dropped onto the sprayed MXene film.

Absorbance measurements of the MXene and MXene@RhB solutions in the range of 300–1350 nm were realized by a SolidSpec-3700 UV-VIS-NIR spectrophotometer (Shimadzu, Kyoto, Japan) using an integrating sphere. Absorbance measurements were performed on liquid solutions in quartz cuvettes. The concentration of MXene solution was 0.63 mg mL^−1^. 5 μL of 1 mM RhB was mixed with either 1.5 mL of MXene solution or deionized water, i.e., the concentration of RhB in the mixture is equal to 0.7 μM. Deionized water was used for baseline correction prior to measurements and also as a reference sample during absorbance measurements. Reflectivity measurements were realized on pristine, MXene-, or RhB-covered filter paper samples.

The SERS experiments were carried out using a WITec alpha 300 R+ confocal Raman microscope. Lasers with wavelengths of 532 and 785 nm and low power (0.2 mW and 0.5 mW, respectively) were employed to excite the Raman signal. A 50× magnification objective (Carl Zeiss 50×/0.8 NA) and a 100 μm diameter optical fiber were used to collect the spectra. Raster scanning images were performed to monitor the spatial homogeneity of the MXenes and analytes signal (20 × 20 μm, 20 × 20 lines). The resulting spectra are an average of 3 regions from the substrates surface. Additionally, single-spot acquisitions were carried out.

## 3. Results and Discussion

### 3.1. Few Layer MXene Nanoflakes

**Figure 1:**
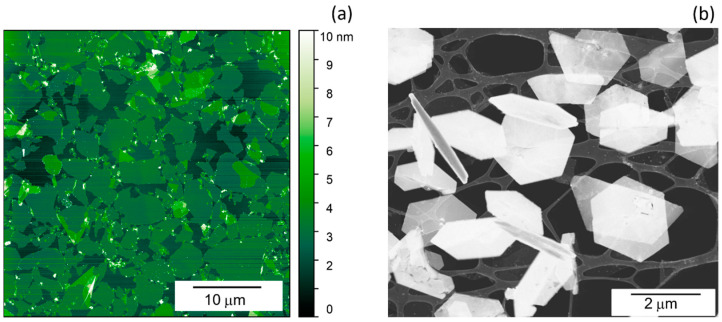
AFM (**a**) and STEM (**b**) image of FL MXene 2D nanoflakes.

AFM and STEM images of the prepared FL Ti_3_C_2_T*_x_* MXene flakes are shown in Figure 1. From the AFM analysis, the mean lateral size and thickness were 4.0 ± 2.7 μm and 1.2 ± 0.2 nm, respectively, confirming the successful delamination process. The nanosheets have smooth morphology, their mean surface roughness value determined by AFM was approximately 0.22 nm. Additionally, a period of 1.32 nm for FL Ti_3_C_2_T*_x_* was determined from the 1st order peak position of the GIXRD pattern (Figure S1).

The chemical composition of Ti_3_C_2_T*_x_* MXenes was inspected by XPS for the drop-casted FL MXene solution on the Si substrate. The survey and C1s, Ti2p, F1s, and O1s region spectra are presented in Supplementary Materials, Figure S2a,b,c,d and e, respectively. The FL MXenes are very clean, which demonstrates very low signal of sp^3^ carbon and relatively high signals related to a Ti_3_C_2_ structure (C1s at ca 283 eV and Ti2p_3/2_ at ca 454–456 eV) [39]. Low signal of Ti^4+^ (Ti2p_3/2_ at ≈ 458.7 eV, together with low signal of F1s at ≈ 686.1 eV, related to Ti-OF/TiF_2_, indicates very low level of oxidation of MXenes on their surface. There are mainly Ti-F and -Ti=O groups on the surface, which are related to stable and non-hydrolyzed Ti_3_C_2_T*_x_* MXene [39]. As explained in [39] the stoichiometry of our MXene structure can be calculated from the signals related to MXene and termination groups after subtracting the organic contamination (C1s signals above 284 eV). In our case, it was Ti_3_C_1.95_F_1.24_O_0.89_ showing a fluorine-rich surface with a relatively low amount of oxygen functionalities (see Table S1).

### 3.2. Few Layer MXene SERS Substrates

**Figure 2:**
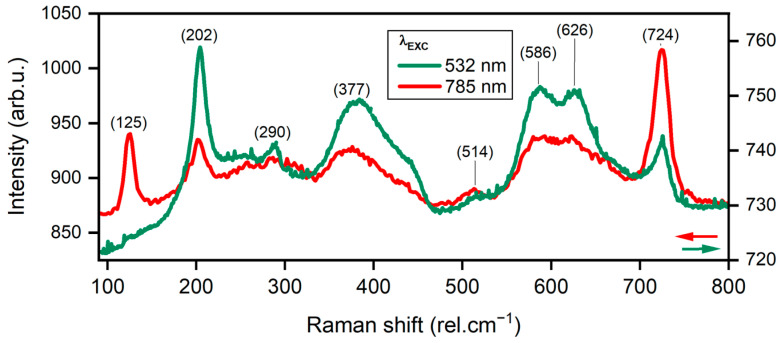
Comparison of Raman spectra of FL MXene film on a glass substrate using excitation laser wavelengths 532 and 785 nm.

The Raman spectra of FL MXenes drop-casted on the glass substrate are shown in Figure 2 for two excitation wavelengths, 532 nm and 785 nm. They are in good correlation with the XPS results. The characteristic Raman peaks from MXenes in accord with published data were identified. The peak at 202 rel.cm^−1^ conforms with out-of-plane vibrations of Ti, O, and C atoms [40]. The peaks at 290, 377, 586, and 626 rel.cm^−1^ are assigned to in-plane shear modes of Ti, C, and the surface-functional groups [40,41]. Small shifts here with regard to the literature can be attributed to different surface termination chemistry. Moreover, the E_g_ in-plane shear mode about the midplane titanium (Ti1) atoms at 125 rel.cm^−1^ is observable only under resonant excitation with a 785 nm excitation laser [42]. Further resonant peaks are at 514 and 724 rel.cm^−1^ belonging to A_1g_ out-of-plane vibrational modes of carbon atoms [40]. The latter is again strongly dependent on the surface termination chemistry [42].

**Figure 3:**
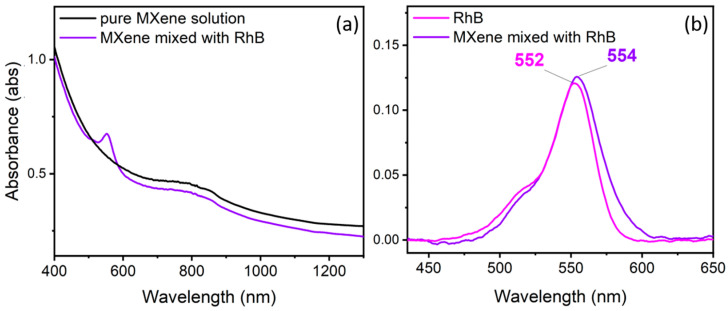
Absorbance characteristics of pristine MXene colloidal solution and RhB mixed with MXenes (**a**). Comparison of baseline subtracted spectra in the region 400–700 nm of the RhB peak in the case of mixtures RhB—deionized water and RhB—MXenes (**b**). The RhB peaks’ maxima are marked.

Figure 3 presents the absorbance spectra of pristine FL Ti_3_C_2_T*_x_* MXene colloidal solution and solution mixed with RhB, and the comparison of RhB absorbance with background subtraction in the region of 400–700 nm. The pure MXene solution shows a broad maximum in the near-IR range, proving the existence of plasmonic properties. Therefore, the possibility of the electromagnetic enhancement mechanism that causes electromagnetic field amplification around the nanoflakes’ atoms after light excitation cannot be excluded. For the RhB–MXene mixture, we identified a shift in the absorbance peak maximum of 2 nm to the infrared region. This indicates the chemisorption of RhB on the MXene surface, which enables the charge transfer processes at the interface of the nanoflakes and the analytes molecules. Similar redshift equal to 12 nm was reported for Rh6G and the Ti-V-C MXenes mixture [32].

The characteristic Raman peaks of RhB in solution are observed between 1300 and 1700 rel.cm^−1^. According to the DFT calculation [43], they could be assigned to the C-C bending at 1355, aromatic C-H bending at 1504, and 1525 and the aromatic C-C bending and C=C stretching at 1644 rel.cm^−1^ [43].

**Figure 4:**
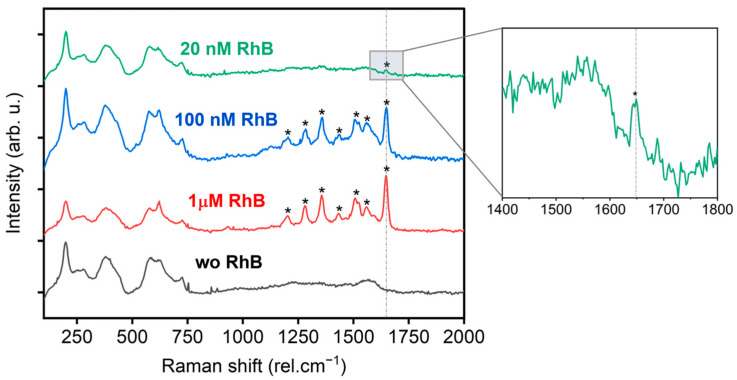
The Raman signal from MXene modified filter paper substrates, with and without deposited RhB, at an excitation wavelength of 532 nm. All spectra underwent background subtraction. Peaks of RhB are marked by asterisk.

The RhB Raman spectra for various concentrations of RhB deposited on the FL MXene substrate are shown in Figure 4 for the 532 nm excitation wavelength. Note, that the RhB and FL Ti_3_C_2_T*_x_* complex showed Raman enhancement only for 532 nm in comparison to 785 nm, which is related to the optical absorption properties of the analyte. The MXene nanosheets were deposited on filtration paper by the VAF method. The LoD of the analyte RhB was determined to be 20 nM. The characteristic peaks of RhB are clearly visible in the Raman spectrum for all analyte concentrations. The spectrum without RhB shows only the characteristic peaks of MXenes. Therefore, the substrates presented in our work surpass the LoD reported so far for molecular dyes (micromolar ranges), showing high potential.

The comparison of reflectivity spectra from filter paper substrates either pristine or covered with MXenes with or without the RhB analyte is shown in Figure S3. A slight redshift can be observed in the case of the spectrum from 100 μM RhB MXene-covered filter paper vs. 100 μM RhB on the pristine paper substrate. A similar shift, however more evident, was demonstrated for absorbance measurements after mixing RhB and MXene solutions as well (Figure 3). This could indicate interaction of MXene nanosheets and RhB molecules on the filter paper substrate.

Different deposition techniques for the preparation of MXene SERS substrates result in different MXene nanosheets assembling during the formation of a film, affecting the morphology of the deposited layer as well as the SERS performance. As the spray coating of MXenes is often used for preparation of SERS substrates (see, e.g., [44,45]), the spray-coated MXene layers were also prepared and analyzed in addition to the VAF MXene layers.

**Figure 5:**
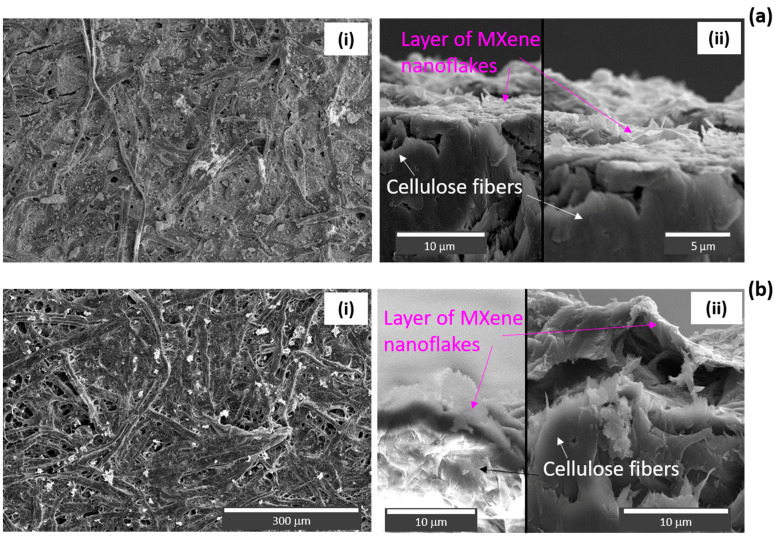
SEM images of VAF (**a**) and spray-coated (**b**) filter paper with MXenes. Images (**i**) show the surface; images (**ii**) show cross sections. High-contrast regions are the result of charging effects. Some of the images are from samples that were sputtered with 5 nm thick Au layer to compensate for this effect.

Figure 5a shows the SEM images of MXene SERS substrates prepared by VAF on filtration paper. Image (i) in Figure 5a presents the typical surface morphology of the VAF-deposited MXene layer on filtration paper. The MXene nanoflakes preferentially copy the underlying surface and the fibrous structure of the underlying paper could be still distinguished. The SEM image of pristine filtration paper is shown in the Supplementary Materials (Figure S4). The layer of MXene nanoflakes in cross section shown in image (ii) on Figure 5a is confirmed by EDS analysis (Figure S5 in the Supplementary Materials). The MXenes form a dense solid layer approximately 1–1.3 μm thick of lying flakes as it is typical for the VAF deposition process. From AFM (see Figure S6 in the Supplementary Materials), we determined a mean square roughness of 56 nm and 52 nm, for pristine and MXene-covered filtration paper, respectively, over an area of 1 × 1 μm^2^. We can conclude that VAF deposition of MXenes does not cause dramatic changes in the mean square roughness of the substrate.

The SEM analysis of the spray-coated MXene layer deposited onto the filtration paper is shown in Figure 5b. The thickness of the layer was close to that of VAF-deposited substrates, i.e., around 1–1.5 μm. While image (i) shows the surface, the layer of MXene nanoflakes in cross section is presented in image (ii), confirmed by EDS analysis (Figure S7 in the Supplementary Materials).

**Figure 6:**
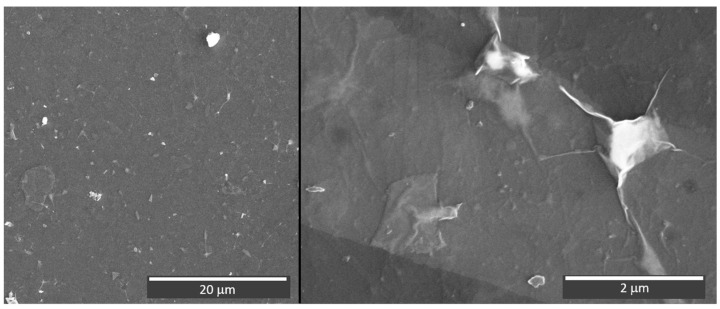
SEM images of spray-coated MXene glass substrate.

The AFM analysis (see Figure S8 in the Supplementary Materials) indicates a higher mean square surface roughness of 122 nm of the spray-coated MXene layer in comparison to the layer deposited by VAF. The higher roughness could point to the tendency to form aggregates of MXenes nanoflakes during the spray coating. To analyze whether the aggregate formation is induced by the rough paper surface (mean square roughness of 56 nm), the glass substrate with a mean square surface roughness of around 1–2 nm was spray-coated under the same deposition conditions. With the aim of analyzing the early stage of the formation of the aggregates during the deposition process, a thin layer of MXenes of approximately 100 nm thick was deposited on the glass substrate. The thickness of the spray-coated layer was determined using profilometry (see Figure S9 in the Supplementary Materials). The SEM image of the deposited layer is shown in Figure 6, revealing the presence of aggregates with an average size of up to several microns (the EDS analysis is presented in Figure S10 in the Supplementary Materials). The AFM analysis (see Figure S11 in the Supplementary Materials) showed that the mean square roughness of spray-coated MXene glass substrates can start at a value of 6.5 nm over a 1 × 1 μm^2^ area, which suggests that the nanoflakes are preferentially lying on the substrates. For areas where the aggregates are located, a mean square roughness value of up to 65 nm was observed. This shows that the formation of aggregates is not influenced by high surface roughness values of the substrate. Instead, the deposition process, such as spray coating with gradual liquid evaporation during drying, encourages particle grouping, leading to aggregate formation.

**Figure 7:**
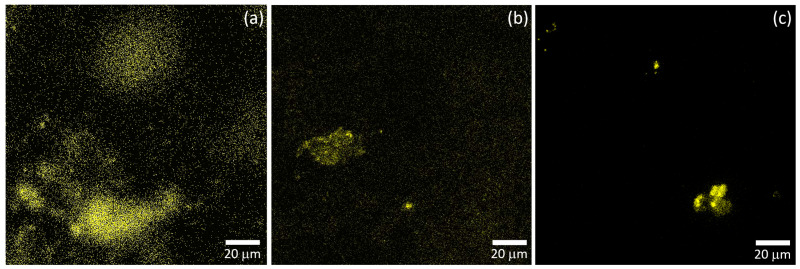
Fluorescence of RhB scanned over samples prepared by VAF on paper (**a**), spray coating on paper (**b**) and spray coating on glass (**c**). The concentration of the deposited RhB was 1 μM.

The effect of the surface morphology on the distribution of analyte over the substrate surface was studied by CLSM. The fluorescence signal from RhB was scanned over a region of equal size for all three substrates, presented in Figure 7. The analyte deposited by VAF on the substrate on which the VAF method was used for preparation, is spread over the entire surface, as shown in Figure 7a. For spray-coated filter paper, the overall uniformity of the analyte distribution decreased, as shown in Figure 7b. For the spray-coated glass substrate where the presence of a large number of aggregates was observed (Figure 6), the analyte molecules are preferentially located in the vicinity of the aggregates, as shown in Figure 7c. The lower uniformity of the analyte distribution for the spray-coated substrates is strengthened by the analyte drop casting. Droplet drying results in unequal distribution of the analyte over the SERS substrate surface. Since the drop dries over 5–10 h, diffusion of the analyte molecules can also be assumed [46].

The data presented demonstrate the advantages of the VAF deposition method for both MXenes and the analyte. In this way, a homogeneous and uniformly dense MXene layer can be prepared. We can calculate a rough estimate of the compactness of the MXene films on the paper surface, based on the dimensions of the created layers (area and thickness of the MXene layer), as well as on the concentration and volume of the MXene solution used. The MXenes’ areal density on the VAF-based and sprayed papers is around 0.2 and 0.06 mg.cm^−2^, respectively. Given that we determined by SEM measurements the same final thickness of the MXene layer in both cases (1–1.5 μm), we can conclude that the VAF-based paper substrates exhibit indeed higher compactness. This in turn could be actually evidence of a highly parallel orientation of the MXene nanosheets to the substrate surface. Moreover, the VAF deposition of the analyte is a type of molecular enrichment approach to increase the affinity of the analyte while achieving increased homogeneity of the distribution of the deposited analyte. The MXene aggregates which could form during the deposition process significantly affect the analyte distribution. We observed increased analyte concentration in the vicinity of the aggregate.

Our observation is underlined by confocal Raman microscopy measurements of the signal of RhB deposited on spray-coated paper and glass, as shown in Figures S12 and S13 in the Supplementary Materials. An LoD of 50 nM has been achieved with spray-coated MXenes on filter paper while the LoD of the spray-coated MXene layer on the glass substrate remains in the micromolar ranges. For the latter, we suspect that the lower concentrations may have been difficult to track due to the clustering effect of the analyte molecules around MXene aggregates. We further hypothesize that the decrease in sensitivity in the case of spray-coated MXene-based paper substrates compared to the substrates prepared using VAF could lie in the absence of molecular enrichment (analyte is drop-casted instead of VAF). To evaluate further the MXene-based paper substrates’ potential as SERSing platforms, we provide comparison for RhB with the LoD of commercially available silver-based ITO substrates (prepared by electrodeposition), as shown in Figure S14 in the Supplementary Materials. These commercial substrates achieved an LoD of 5 nM, showcasing the VAF-deposited MXenes-based paper substrates in a very competitive light.

**Figure 8:**
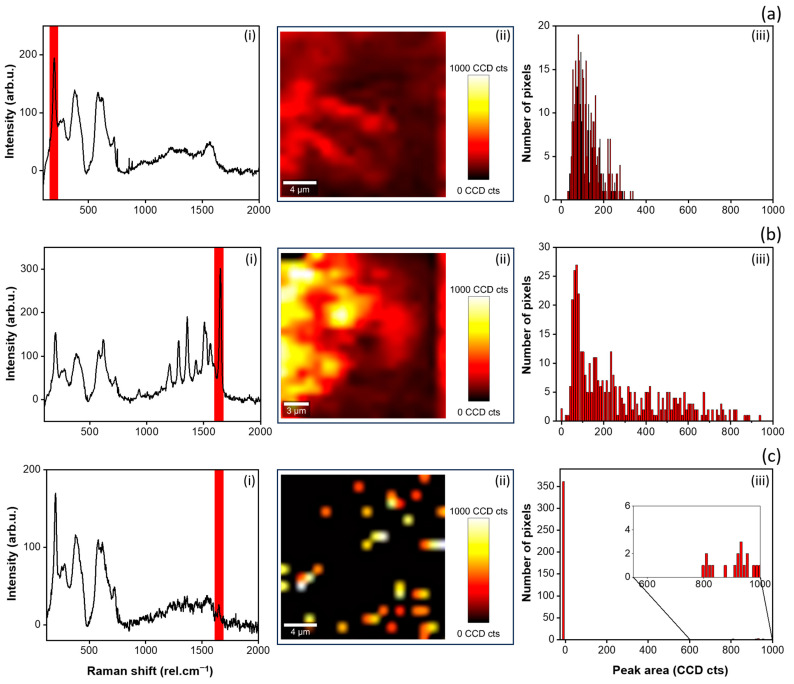
Distribution of the MXenes (**a**) and analyte RhB (**b**,**c**) over the filtration paper-based substrates prepared and deposited by VAF. (**b**,**c**) presents the RhB concentrations 100 and 20 nM, respectively. Images (**i**) show examples of the measured spectra from one point of the scanned 20 × 20 μm^2^ area. The fitted areas under the significant peaks are marked with red rectangular shapes. Images (**ii**) show the resulting color maps, representing the peaks’ areas (peak at 202 rel.cm^−1^ for MXenes, peak at 1646.5 rel. cm^−1^ for RhB). Images (**iii**) show the corresponding histograms, i.e., the peak area as function of number of pixels over the scanned area.

The uniformity of the MXene layer and the distribution of RhB analyte over the MXene layer was further investigated by Raman peak intensity mapping for VAF-deposited MXenes and analyte substrates. Figure 8 presents the distribution of the MXene flakes (a) and RhB molecules (b, c) over the prepared substrate’s surface. The highlighted spectral region on the graphs (i) (width 75 rel.cm^−1^) was fitted over the 20 × 20 μm^2^ scanned area. The area under the fitted peaks was extracted. Images (ii) show the resulting area, representing the distribution of MXenes or RhB in a color map, with corresponding histograms, as shown in images (iii). It is evident that the MXenes cover the whole inspected region homogenously (Figure 8a(ii)), with small variations in the peak areas (Figure 8a(iii)). Figure 8b,c present the distribution of the analyte RhB deposited with 100 and 20 nM concentration, respectively. Despite the low concentration, at 100 nM we see almost no region without the presence of the RhB peak (the number of pixels with an area under the peak equal to zero is small). On the other side, the results on RhB with a 20 nM concentration reveal less homogenous distribution of the signal. This could be attributed to the low concentration, i.e., smaller number of analyte molecules on the surface, although seemingly scattered randomly.

One can remark that we observed a shift in the present work for the RhB peak belonging to the aromatic C–C bending and C=C stretching peak, with a theoretically determined position of 1644 rel.cm^−1^ [43]. This shift underlines the chemical enhancement effect, that supports the conclusions drawn from the absorbance and reflectivity measurements, where the RhB peak shifted after mixing with MXene solution, and after deposition on MXene-covered filtration papers (see Figure 3 and Figure S3). These shifts can be explained by changes in the vibrational modes induced by chemical interactions between the substrate molecules and the analyte [32]. The adsorption of the analyte molecules on the MXenes surface could form chemical bonds, which can serve as charge transfer channels to facilitate the redistribution of the electron cloud around the RhB molecules and MXenes. For comparison, the Ag-based ITO commercial substrates showed a redshift of only 0.7 rel.cm^−1^, which is in accordance with the literature, as noble metals are known to have a more important electromagnetic than a chemical enhancement factor. MXene-filtered paper substrates displayed a red shift of around 2.5 rel.cm^−1^.

## 4. Conclusions

In summary, this study introduced a novel SERS platform that utilizes vacuum-assisted filtration (VAF) to prepare highly sensitive paper-based Ti_3_C_2_T*_x_* MXene substrates for the detection of Rhodamine B dye. The VAF method allowed for the deposition of both the MXene nanoflakes and the analytes, resulting in a compact and uniform layer without aggregates. This approach led to significantly improved sensitivity, enabling the detection of Rhodamine B down to 20 nM concentrations, surpassing previous reports. Filter paper and glass substrates spray-coated with MXenes were prepared for comparison. The substrates were characterized in detail with emphasis on their morphological properties in relation to their SERS performance. For spray deposition of MXenes, the formation of aggregates was observed. This process starts from the early stage of the deposition and results in larger surface roughness and complex morphology of the spray-coated MXene layers, which could deteriorate their SERS performance in comparison to the VAF-deposited substrates. The presence of aggregates lowers the uniformity of the analyte distribution on the spray-coated SERS substrate. The effect is strengthened by the analyte drop casting. Droplet drying results in unequal distribution of the analyte over the SERS substrate surface. Since the drop could dry over 5–10 h, diffusion of the analyte molecules can be assumed. This study highlights the potential of VAF-based MXene substrates for sensitive SERS applications.

## Data Availability

The data and materials that support the findings of this study are available from the corresponding author upon reasonable request.

## Supplementary Materials

The following supporting information can be downloaded at: https://www.mdpi.com/article/10.3390/ma17061385/s1, Figure S1: GIXRD pattern of the of FL Ti_3_C_2_T*_x_* MXene showing three MXene orders. The number 1.32 nm MXene period derived from the 1st order peak position; Figure S2: XPS of 2D MXene of (a) survey, (b) C1s, (c) Ti2p, (d) F1s and (e) O1s region; Figure S3: Reflectivity measurements of filter paper substrates. The comparison of pristine and MXene covered filter papers (a). The comparison of the signal after background subtraction of reflectivity in the case of 100 µM RhB on pristine and MXene covered filter papers (b). RhB on filter paper (c). RhB on MXene covered filter paper substrates (d); Figure S4: SEM images of pristine filtration paper, from the surface (a) and cross section (b); Figure S5: EDS SEM analysis from the cross section of VAF MXene on filter paper, illustrating the signal from the paper (pt 1) vs. the MXene layer (pt 2). The samples were sputtered with 5 nm thick Au layer to compensate charging effects during cross section measurements, which explains the presence of Au in the signal; Figure S6: AFM images of pristine (a) and MXene covered filtration paper (b); Figure S7: EDS SEM analysis from the cross section of spray coated MXene on filter paper, illustrating the signal from the paper (pt 2) vs. the MXene layer (pt 1). The samples were sputtered with 5 nm thick Au layer to compensate charging effects during cross section measurements, which explains the presence of Au in the signal; Figure S8: AFM image of spray coated MXene on filtration paper; Figure S9: Profilometry result of spray coated MXene glass substrate. The valleys correspond to scratched surfaces, i.e., where the MXene layer was removed to determine its thickness; Figure S10: EDS SEM analysis of the presence of MXenes on the spray coated glass substrates. The darker areas represent thicker regions of the MXene layer; Figure S11: AFM image of spray coated MXene glass substrate; Figure S12: Raman spectra of RhB drop casted on spray coated MXene on filter paper substrates of different concentrations (1 µM, 100 nM, 50 nM, 20 nM). Excitation wavelength 532 nm. All spectra underwent background subtraction. Although we see an indication for the development of RhB peaks even at 20 nM, no clear peak could be distinguished for none of the scanned areas; Figure S13: Raman spectra of RhB drop casted on blank glass and spray coated MXene glass substrates of different concentrations (0, 1 mM, 500 µM, 1 µM). Excitation wavelength 532 nm. All spectra underwent background subtraction; Figure S14: Raman spectra from commercial SERS ITO substrates, with and without Rhodamine B drop casted on the surface. Excitation wavelength 532 nm. All spectra underwent background subtraction; Table S1: Apparent surface chemical composition of FL MXene as determined by XPS.
