# Supplementary material for: SERS Performance of Ti3C2Tx MXene-Based Substrates Correlates with Surface Morphology
Source: Materials (Basel). 2024 Mar 18;17(6):1385. doi: 10.3390/ma17061385 (PMC10971885; doi:10.3390/ma17061385)
Supplement: Supplementary file 1 [file materials-17-01385-s001.zip › materials-2872978-supplementary.pdf]

SUPPLEMENTARY INFORMATION

SERS performance of $Ti_3C_2T_x$ MXene-based substrates correlates with surface morphology

Farnoush Salehtash,[a] Adriana Annušová,[abc] Anastasiia Stepura,[c] Yaryna Soyka,[c]
Yuriy Halahovets,[a] Monika Hofbauerová,[ab] Matej Mičušík,[c] Mário Kotlár,[d] Peter Nádaždy,[ae]
Paweł Albrycht,[f] Peter Šiffalovič,[ab] Matej Jergel,[ab] Mária Omastová,[c] and Eva Majková[ab]*

[a.] *Institute of Physics, Slovak Academy of Sciences, Dúbravská cesta 9, 845 11 Bratislava, Slovakia*

[b.] *Centre for Advanced Materials Application, Slovak Academy of Sciences, Dúbravská cesta 9, 845 11 Bratislava, Slovakia*

[c.] *Polymer Institute, Slovak Academy of Sciences, Dúbravská cesta 9, 845 41 Bratislava, Slovakia*

[d.] *Centre for Nanodiagnostics of Materials, Slovak University of Technology in Bratislava, Vazovova 5, 812 43 Bratislava, Slovakia*

[e.] *Institute of Electrical Engineering, Slovak Academy of Sciences, Dúbravská cesta 9, 841 04 Bratislava, Slovakia*

[f.] *Institute of Physical Chemistry, Polish Academy of Sciences, Kasprzaka 44/52, Warsaw, 01-224, Poland*

* *eva.majkova@savba.sk*

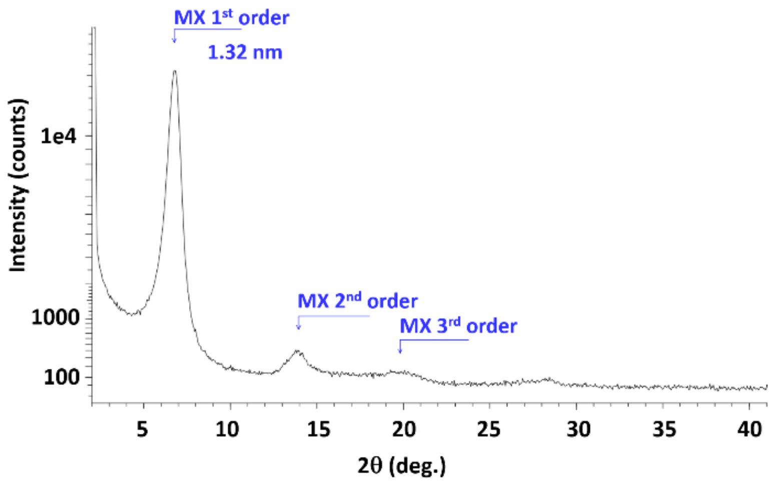

Figure S1 GIXRD pattern of the of FL $Ti_3C_2T_x$ MXene showing three MXene orders. The number 1.32 nm MXene period derived from the 1st order peak position.

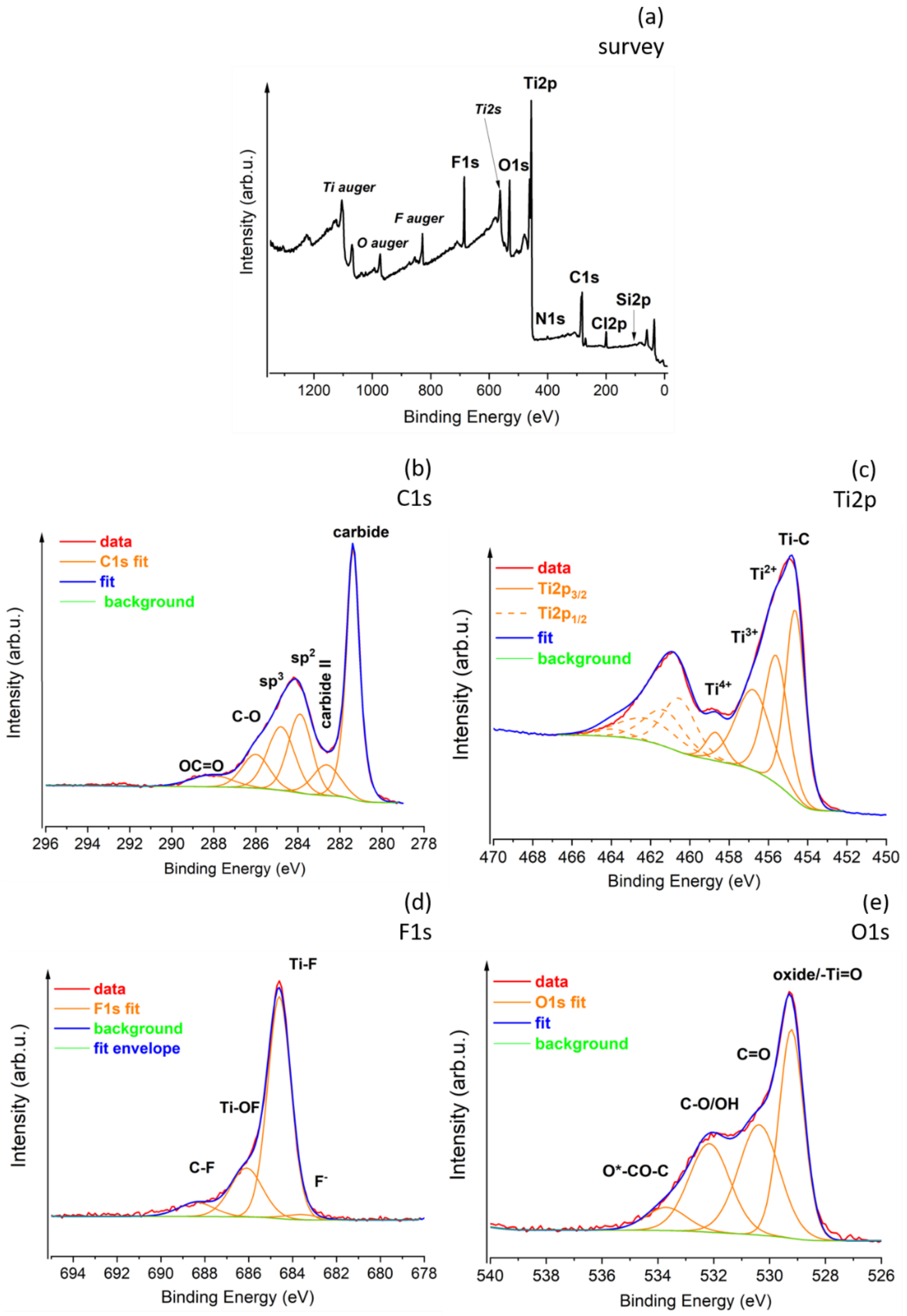

Figure S2 XPS of 2D MXene of a) survey, b) C1s , c) Ti2p, d) F1s and e) O1s region.

Table S1 Apparent surface chemical composition of FL MXene as determined by XPS.

	Surface chemical composition (at.%)				
	C 1s<br>Ti_3C_2/I/II/sp^3/CO/<br>OCO	O 1s<br>ox/I/II/III	Ti 2p<br>Ti_3C_2/I/II/III	F 1s<br>F^-/TiF/OTiF/<br>CF	Cl2p/Si2p<br>N 1s
FL-<br>MXene	36.3<br>13.8/3.2/7.1/6.7/3.6/<br>1.9	19.0<br>6.9/6.0/4.8/1.3	27.7<br>10.1/8.4/7.7/1.5	11.9<br>0.3/8.3/2.5/0.8	2.7/1.0/<br>1.4

C1s: I – carbide signal, II – sp^2 signal
O1s: I – C=O, II-C-O/-OH, III – O*-CO-C
Ti2p: I – Ti^{2+}, II – Ti^{3+}, III - -Ti=O

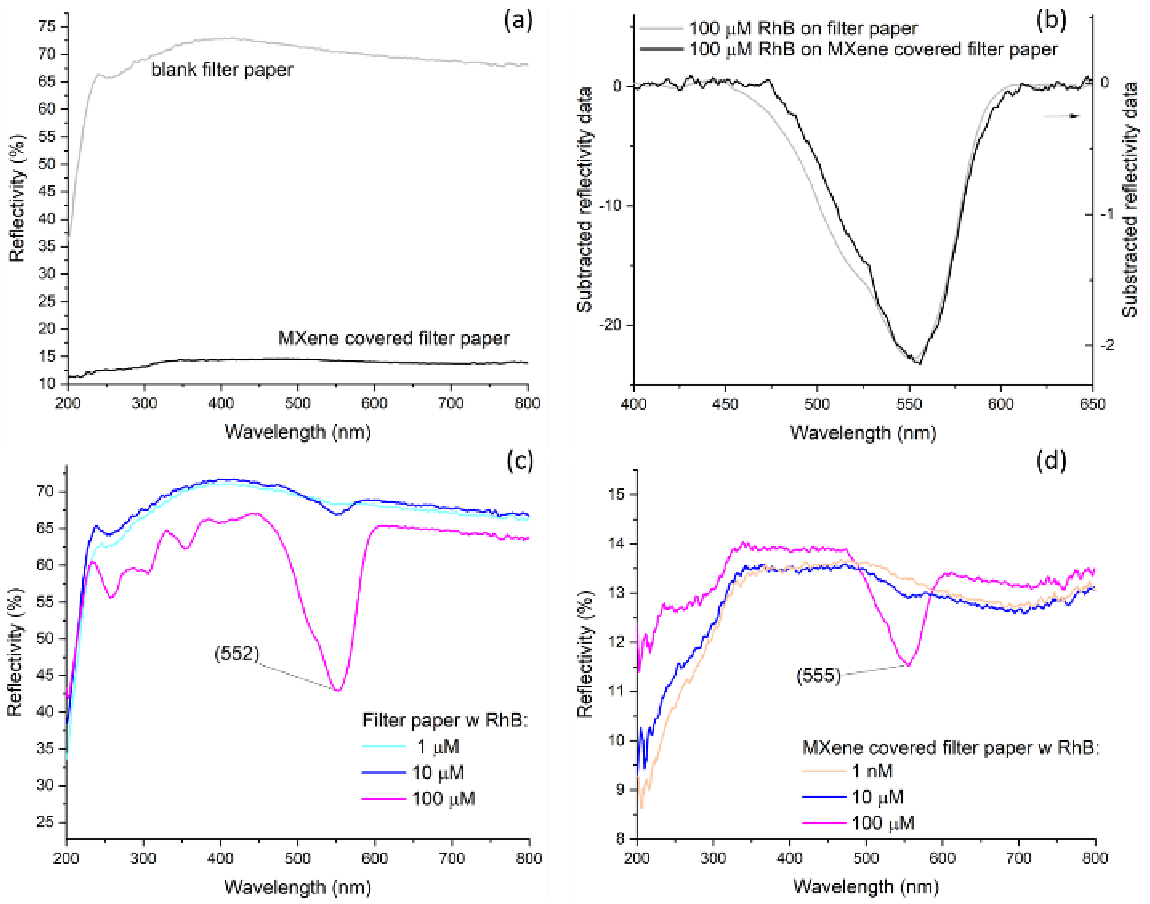

Figure S3 Reflectivity measurements of filter paper substrates. The comparison of pristine and MXene covered filter papers (a). The comparison of the signal after background subtraction of reflectivity in the case of 100 µM RhB on pristine and MXene covered filter papers (b). RhB on filter paper (c). RhB on MXene covered filter paper substrates (d).

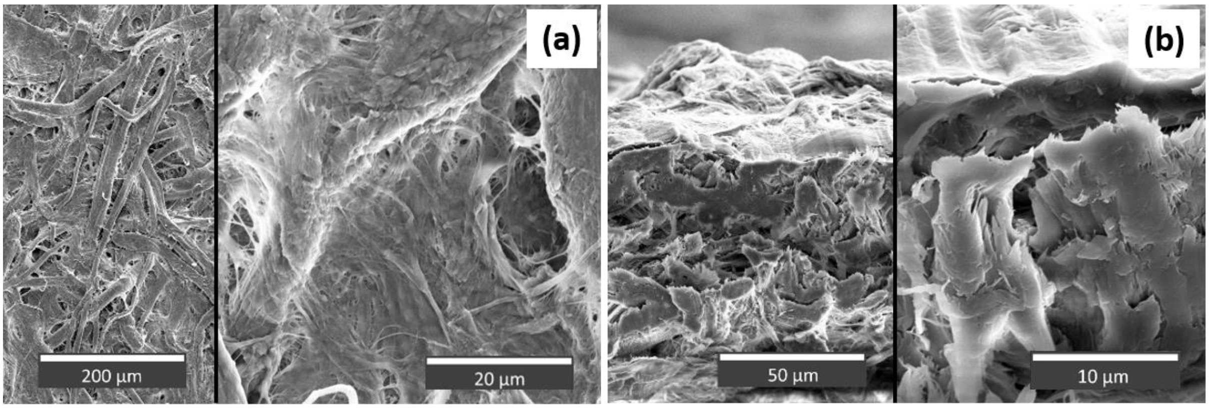

Figure S4 SEM images of pristine filtration paper, from the surface (a) and cross section (b).

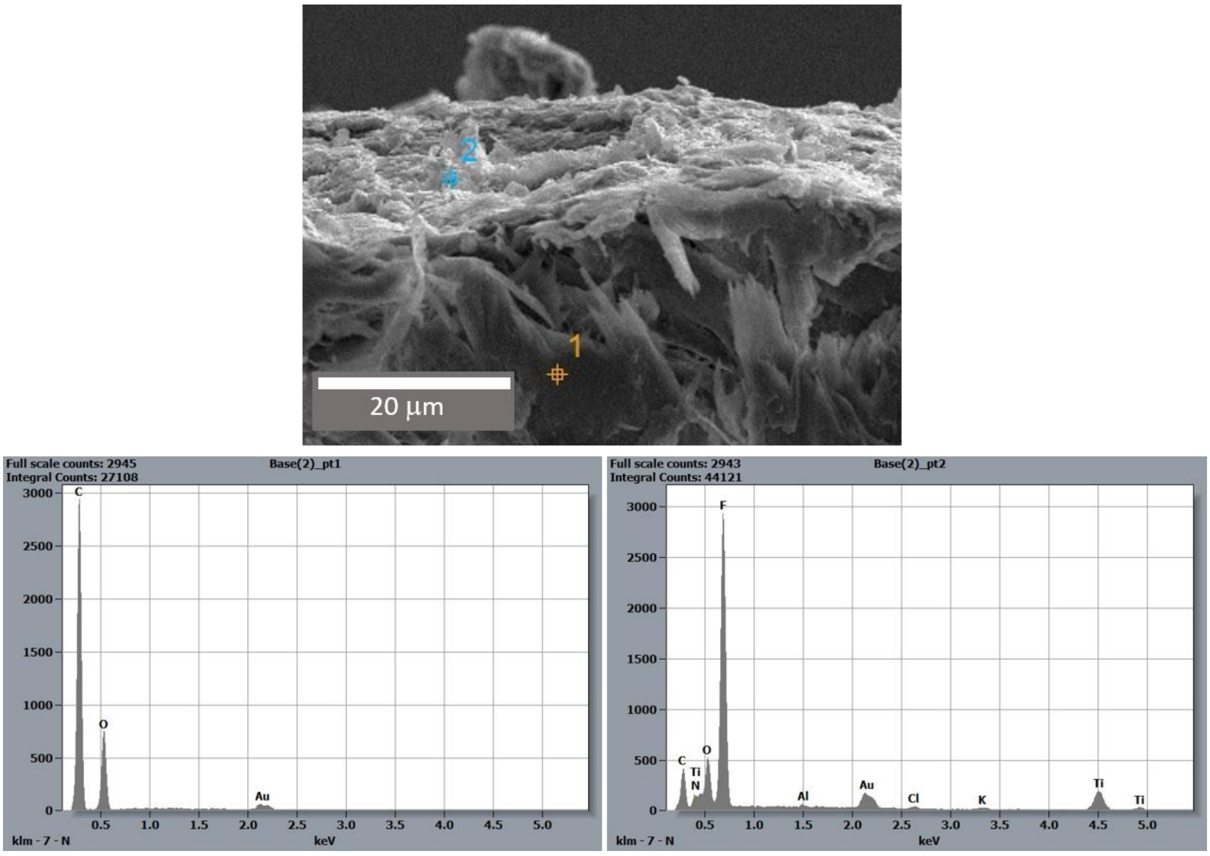

Figure S5 EDS SEM analysis from the cross section of VAF MXene on filter paper, illustrating the signal from the paper (pt 1) vs the MXene layer (pt 2). The samples were sputtered with 5 nm thick Au layer to compensate charging effects during cross section measurements, which explains the presence of Au in the signal.

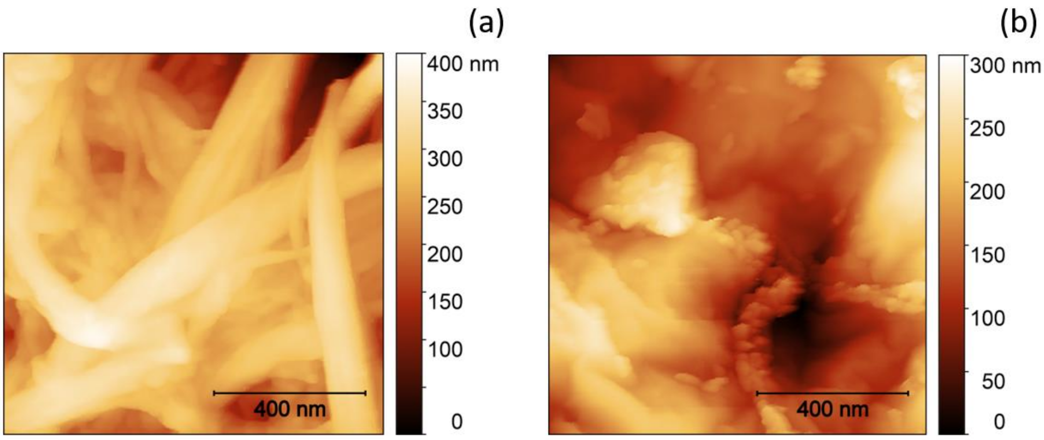

Figure S6 AFM images of pristine (a) and MXene covered filtration paper (b).

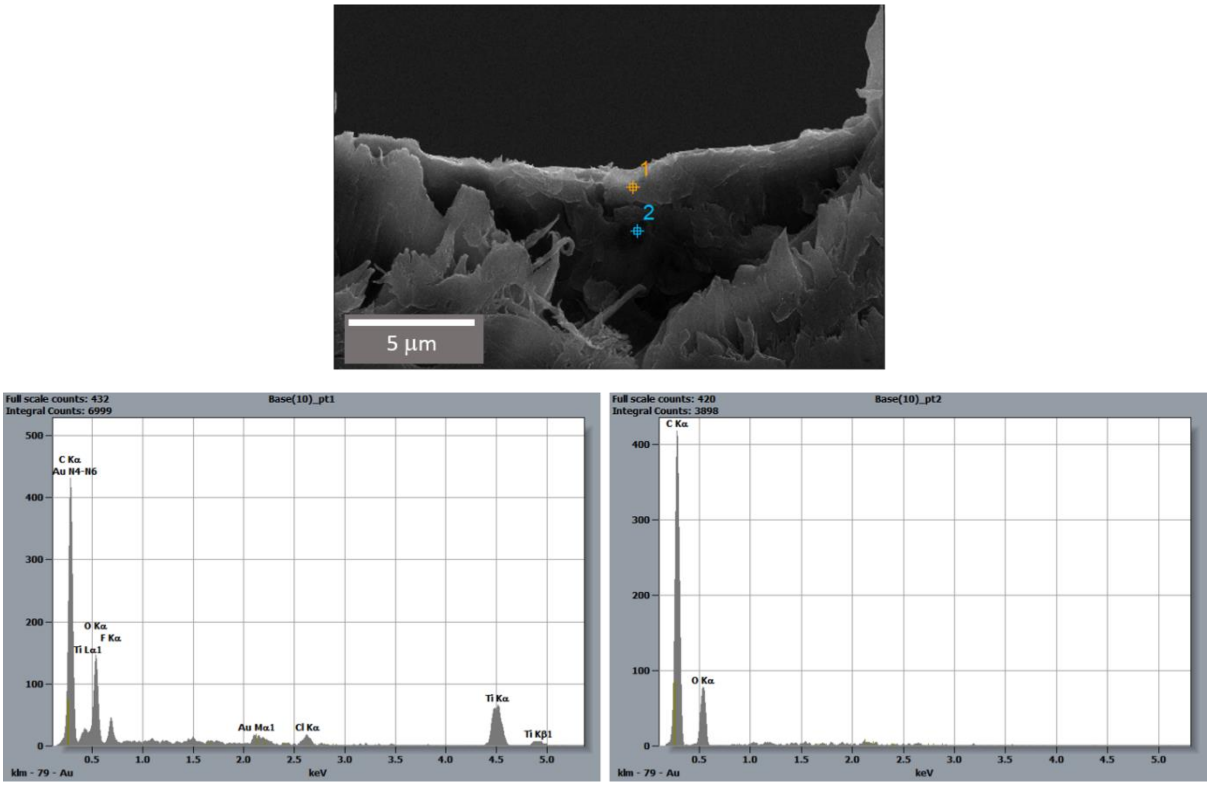

Figure S7 EDS SEM analysis from the cross section of spray coated MXene on filter paper, illustrating the signal from the paper (pt 2) vs the MXene layer (pt 1). The samples were sputtered with 5 nm thick Au layer to compensate charging effects during cross section measurements, which explains the presence of Au in the signal.

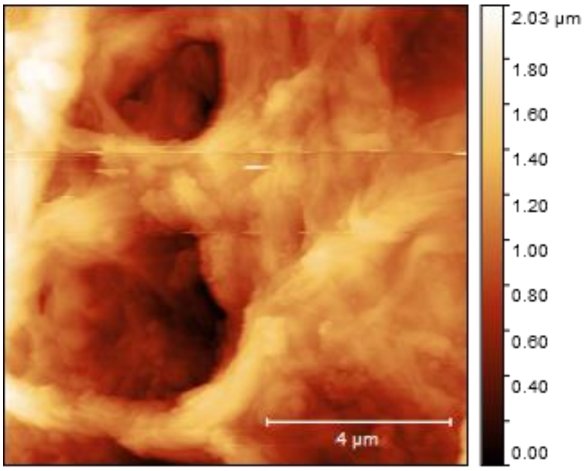

Figure S8 AFM image of spray coated MXene on filtration paper.

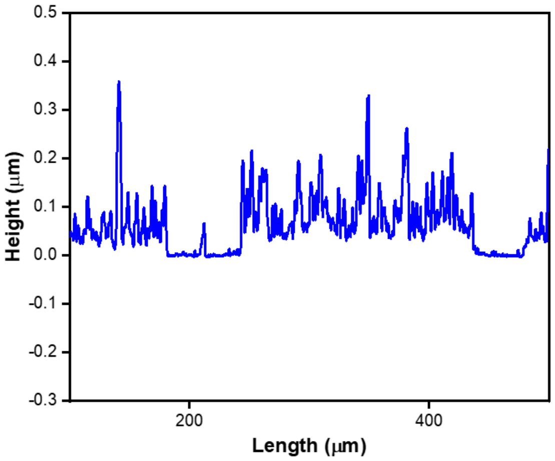

Figure S9 Profilometry result of spray coated MXene glass substrate. The valleys correspond to scratched surfaces, i.e. where the MXene layer was removed to determine its thickness.

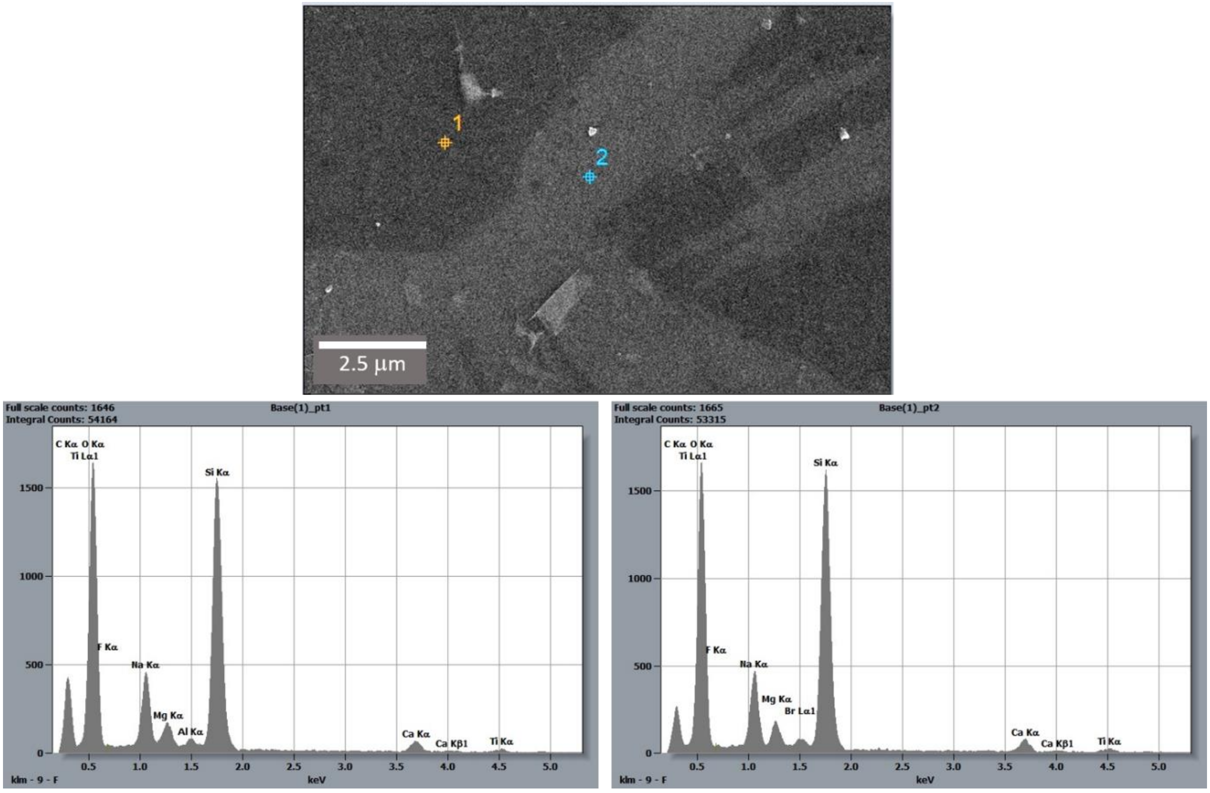

Figure S10 EDS SEM analysis of the presence of MXenes on the spray coated glass substrates. The darker areas represent thicker regions of the MXene layer.

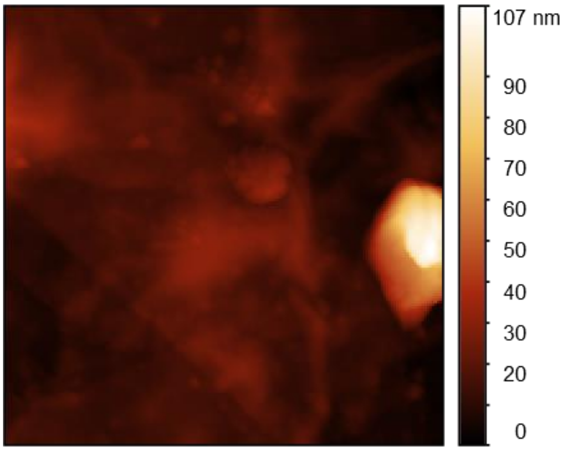

Figure S11 AFM image of spray coated MXene glass substrate.

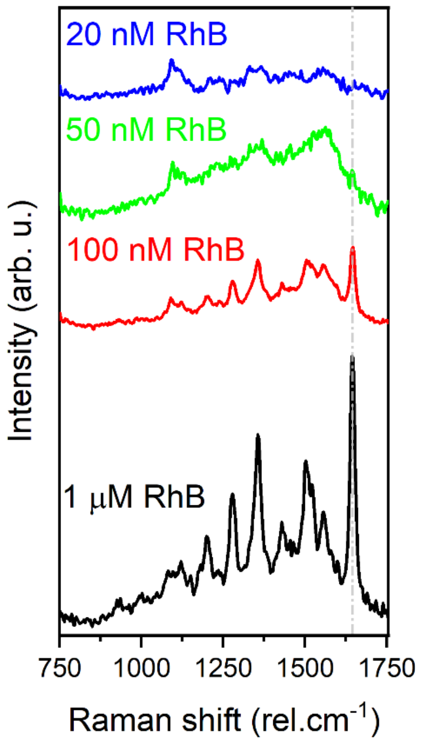

Figure S12 Raman spectra of RhB drop casted on spray coated MXene on filter paper substrates of different concentrations (1 µM, 100 nM, 50 nM, 20 nM). Excitation wavelength 532 nm. All spectra underwent background subtraction. Although we see an indication for the development of RhB peaks even at 20 nM, no clear peak could be distinguished for none of the scanned areas.

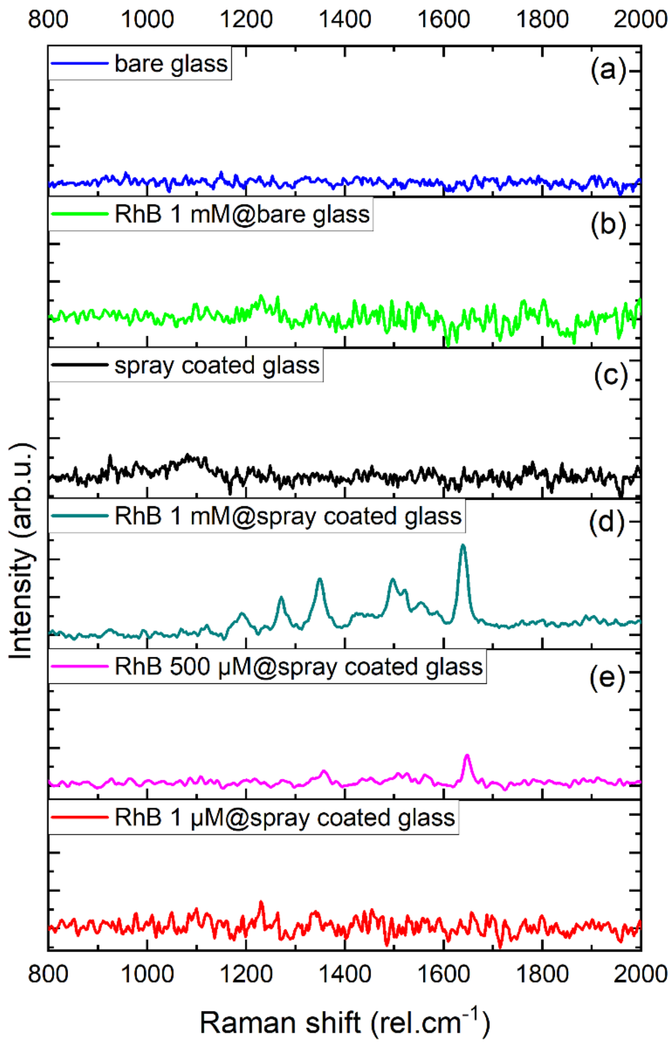

Figure S13 Raman spectra of RhB drop casted on blank glass and spray coated MXene glass substrates of different concentrations (0, 1 mM, 500 μM, 1 μM). Excitation wavelength 532 nm. All spectra underwent background subtraction.

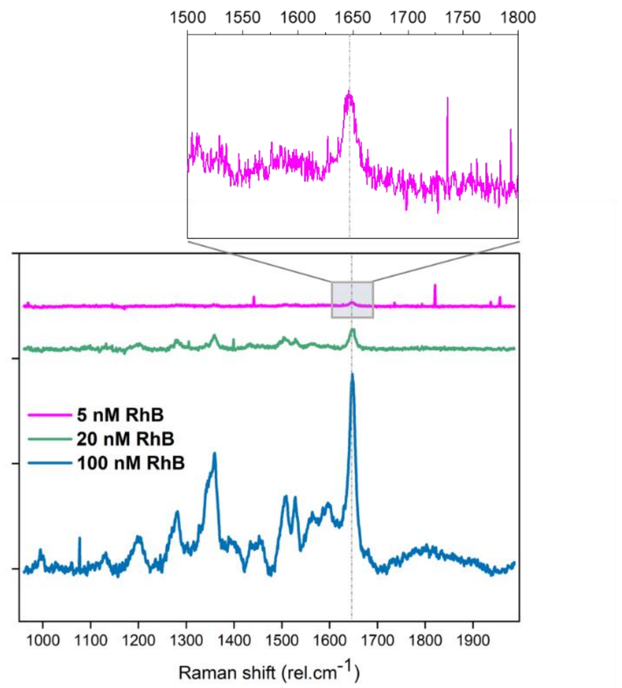

Figure S14 Raman spectra from commercial SERS ITO substrates, with and without Rhodamine B drop casted on the surface. Excitation wavelength 532 nm. All spectra underwent background subtraction
